# Effectiveness of a theory-based educational intervention on work-related musculoskeletal disorders preventive behaviors among assembly-line female workers: a study protocol for a randomized controlled trial

**DOI:** 10.1186/s13063-023-07391-0

**Published:** 2023-06-05

**Authors:** Zakieh Sadat Hosseini, Sedigheh Sadat Tavafian, Omran Ahmadi, Reza Maghbouli

**Affiliations:** 1 Department of Health Education, Faculty of Medical Sciences, Tarbiat Modares University, Tehran, Iran; 2 Department of Occupational Health, Faculty of Medical Sciences, Tarbiat Modares University, Tehran, Iran; 3 Hasheminejad Hospital, School of Medicine, Iran University of Medical Sciences, Tehran, Iran

**Keywords:** Musculoskeletal disorders, Workers, Intervention, Female, Behavior

## Abstract

**Background:**

The use of preventive behaviors of musculoskeletal disorders (MSDs) requires proper training, which leads to correct decisions regarding maintaining postures at work and performing stretching exercises. Due to very repetitive work, applying manual force, improper postures, and static contractions of proximal muscles, assembly-line female workers suffer from musculoskeletal pains. It is assumed that structured and theory-based educational intervention using a learning-by-doing (LBD) approach may increase the preventive behaviors against MSDs and reduce the consequences of these disorders.

**Methods:**

This randomized controlled trial (RCT) will be conducted in three phases: phase 1: validation of the compiled questionnaire, phase 2: determining the social cognitive theory (SCT) constructs that predict the preventive behaviors of MSDs in assembly-line female workers, and phase 3: designing and implementing the educational theory. The educational intervention is based on the LBD approach, and the study population includes assembly-line female workers in electronic industries of Iran, who are randomly divided into two intervention and control groups. The intervention group received the educational intervention in the workplace and the control group does not receive any intervention. The theory-based educational intervention includes evidence-based information along with pictures, fact sheets, and published literature about a good posture at work and the need to perform proper stretching exercises. The educational intervention aims to improve the knowledge, skills, self-efficacy, and intention of assembly-line female workers to adopt preventive behaviors of MSDs.

**Discussion:**

The present study will evaluate the effects of maintaining a good posture at work and performing stretching exercises on the adherence to preventive behaviors of MSDs among assembly-line female workers. The developed intervention is easily implemented and evaluated in a short period of time based on the improved score of the rapid upper limb assessment (RULA) method and the mean score of adherence to stretching exercises and can be provided by a health, safety, and environment (HSE) expert.

**Trial registration:**

ClinicalTrials.gov IRCT20220825055792N1. Registered on 23 September 2022 with the IRCTID.

## Background

Work-related musculoskeletal disorders (WMSDs) are one of the main causes of activity limitation among the working population, the second most common work-related disease, and the fourth leading cause of health-related costs worldwide [1–4]. The high prevalence of these disorders and the need to control risk factors have been mentioned in some studies in recent decades [5–8]. Carrying out repetitive movements and static and improper postures at work and applying manual force are some occupational risk factors for WMSDs among assembly-line workers [9, 10]. WMSDs have led to absenteeism from the work and increased costs and human injuries and are considered as the main cause of disability and impose a significant financial burden on the health care system, individuals, and social care systems in developed and developing countries [11–14].

Ergonomic interventions and corrective measures are standard approaches to prevent WMSDs and increase productivity in the workplace; however, certain factors such as background factors, incorrect interventions, ineffective stakeholder participation, and poor ergonomic analysis can lead to unsuccessful interventions [15]. High-quality ergonomic interventions and exercise at the workplace help to prevent or control WMSDs symptoms [16]. According to the evidence, if workplace exercises are not combined with complementary ergonomic interventions, they will not be very effective [17].

One of the appropriate methods for predicting the risk of WMSDs in various tasks is the rapid upper limb assessment (RULA) [18]. The traditional scoring system of RULA has low sensitivity to input variables, and it is difficult to show the effectiveness of ergonomic interventions in it, while a single value in RULA can belong to several sets with different degrees [19]. This method is able to predict the effectiveness of various interventions in reducing the risk of WMSDs [20]. Despite the benefit of using RULA in measuring the ergonomic behaviors, applying theory-based interventions are recommended in previous studies. As it was argued, individual and environmental factors and maintaining correct sitting postures at work are among the effective factors in preventing WMSDs [21]. Therefore, one of the related theories is social cognitive theory (SCT), which considers processes of cognitive, environmental, and behavioral patterns together and recommends strategies to increase behavioral ability, self-efficacy, and expected outcomes [22]. Training can be more effective when participants are made to practice the recommended behavior and exercises are presented to them compared to the time when a lecture on the recommended behavior is given to them [23]. The learning by doing (LBD) approach is a type of active learning that leads to greater understanding and participation of people in the learning process [24]. Previous studies have used educational theories in their intervention process even in non-industrial environments [25–28], whereas most of the studies that reported the effectiveness of intervention on workers’ performance in the workplace did not use a theory-based or structured training approach [29, 30]. Although the effectiveness of SCT in improving the preventive behaviors of WMSDs has been reported in some studies [31, 32], the effectiveness of this theory in adhering to the recommended behaviors in assembly-line workers that are exposed to many risk factors for WMSDs has not been investigated efficiently [10, 33].

In addition to the direct impact on workers’ health and work disability, sickness absence, disability pensions, loss of productivity, disruption of women’s social roles, and their functional limitation due to WMSDs have negative effects on their quality of life and impose a major socio-economic burden; thus, it is known as a challenge in the work environment [34, 35]. Since health education is one of the main factors for creating health promotion in the primary health care system [36], it is important for researchers to demonstrate the role of theory-based education with an LBD approach in female assembly workers, as it improves clinical practice and scientific research. Furthermore, HSE experts can easily implement structured and theory-based training using the LBD approach in the industry, which will be of great assistance to the health system. Therefore, investigating the theory-based educational intervention of assembly-line female workers using the LBD approach can be an appropriate intervention to encourage them to use preventive behaviors of WMSDs that should be tested. The present article explains the protocol of our study with the aim of determining the effect of SCT-based ergonomic intervention on preventive behaviors of MSDs in assembly-line female workers.

## Method

### Study design

In this protocol, a double-blind, a single-center superiority randomized control trial (RCT) (control: intervention = 50%:50%), parallel controlled with voluntary participation, and 6 months follow-up will be done.

### Study setting

The present study will be conducted in Khorasan-Razavi province, located in the northeast of Iran. Iran is a country with more than 80 million populations, which is divided into 31 provinces. The first level of country subdivisions of Iran is the provinces [37]. Eligible participants will be selected from electronic industries. The study protocol approved by the Ethics Committee of Tarbiat Modares University and registered in the Iranian Registry of Clinical Trials with the number IRCT20220825055792N1. The enrollment, interview, intervention, and evaluation schedule is shown in Table 1. This protocol was developed and reported according to the Standard Protocol Items: Recommendations for Interventional Trials (SPIRIT) [38], and the clinical trial will be conducted and reported in accordance with the consolidated standards of reporting trials (CONSORT). To achieve the objective of the study, the following three phases will be considered (Table 2).

**Table 1:**
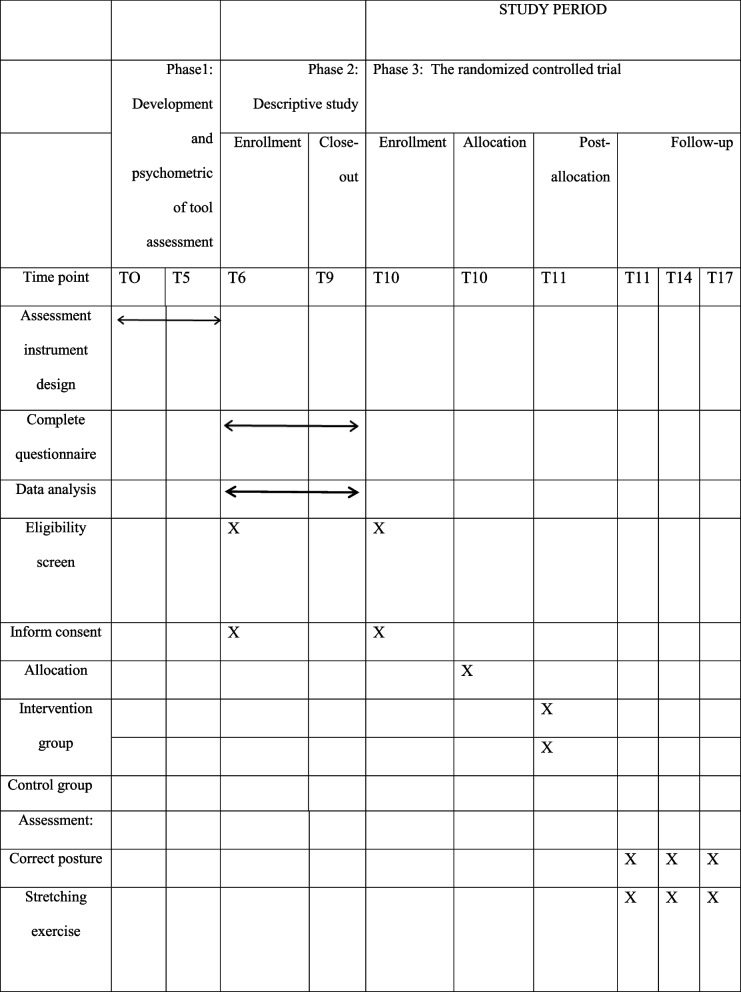
Schedule of enrolment, interviews, intervention, and assessment of the Ergonomic Intervention trial, following the Standard Protocol Items Recommended for Clinical Trials (SPIRIT) guidelines

**Table 2:** The study overview

Phases and participant	Aim	Methods
Phase 1: Development and psychometric of tool assessment	Validation and reliability of the questionnaire and preparation of the final questionnaire	By the research team and expert panel through face and content validity, exploratory factor analysis, reliability, and impact score
Phase 2: Descriptive study		
Determining the most important predictive structures	Identifying the most important predictive structures of social cognitive theory in applying recommended behaviors	Based on a cross-sectional study
Phase 3: The randomized controlled trial		
Design the educational interventional	Step1: Set the educational content	Based on phase 2
Implementation of the intervention	Step2: Transfer concepts to the intervention group	Based on step 1in phase 3
Educational program evaluation	Step3: Investigating the effect of the educational program in the intervention group	Fuzzy RULA method, researcher-made questionnaire, Nordic questionnaire

### Phase 1: Development and psychometric assessments

The researcher-made questionnaire will be designed based on the SCT constructs after reviewing student theses, articles, and similar questionnaires, and then the primary instrument for analyzing the psychometric properties will be prepared.

### Phase 2: Descriptive study

To assess WMSD prevalence, the researcher-made questionnaire and the Nordic questionnaire [39] will be completed in a self-reported manner by the assembly-line female workers. Afterwards, the most important predictors of preventive behaviors of MSDs will be determined after entering data into the Bayesian network [20].

### Phase 3: The randomized controlled trial

**Fig. 1:**
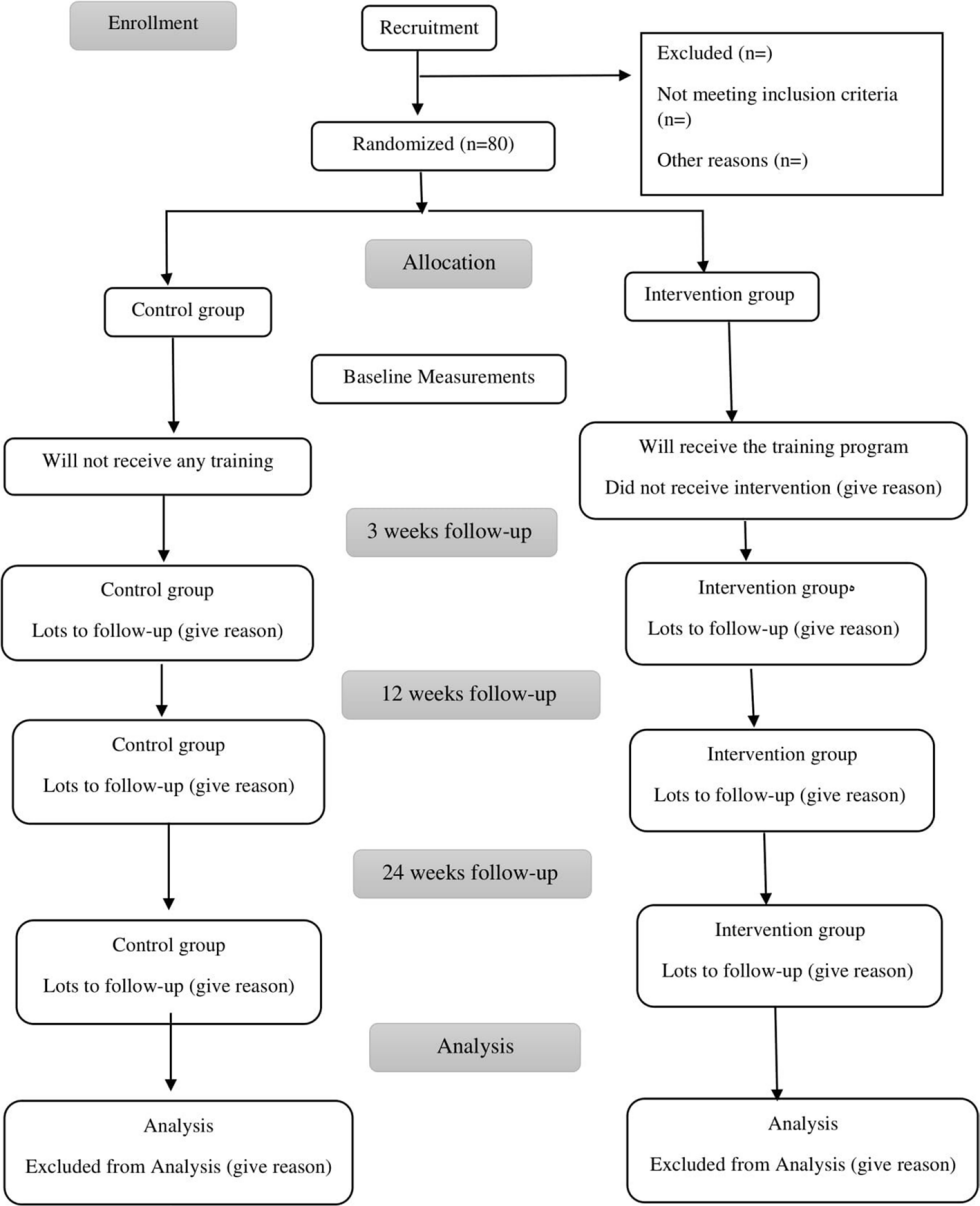
The flow chart of the randomized controlled protocol

Randomized controlled trials (RCTs) are accurate tools for investigating cause-and-effect relationships between an intervention and an outcome because randomization balances participant characteristics between groups and allows any differences in outcome to be attributed to the study intervention [40]. The flow chart of the randomized controlled protocol is shown in Fig. 1

### Eligibility criteria

In an analytical study on Iranian assembly-line female workers in 2020, the mean ± SD of the participants’ age was reported as 28.3 ± 1.81 years, and more than 60% of women reported pain in the shoulder, wrist, and back area [41]. Considering that, the present intervention will be on assembly-line female workers, and it is intended to include the majority of women in the study; therefore, the eligible age range was selected above 20 years. Assembly-line female workers, having literacy and the ability to participate in the program for at least 6 months, are among the inclusion criteria. Exclusion criteria also included being pregnant, being prohibited from doing stretching exercises by the doctor, a change in the workplace, lack of consent to continue participating, and becoming pregnant during the study. Participants will report potential adverse events that may occur during the trial.

### Intervention

The intervention protocol focuses on correcting workers’ posture based on RULA by creating a fuzzy system that is helpful in most workplaces, where a large number of workers are exposed to WMSDs due to poor posture at work [42]. Exercises also include stretching and strengthening exercises of the upper limb, focusing more on the neck, hand, and wrist joints. The intervention program will be developed based on the most important predictive constructs of SCT from phase 2 of the study. The recruitment process will continue until the required sample size is reached. The researcher-made questionnaire, the standardized Nordic questionnaire, and the RULA method will be completed prior to the intervention. The intervention arm includes providing an educational intervention through the LBD process during assembly work. This intervention focuses on awareness and acquiring skills to maintain a good body posture at work and perform appropriate stretching exercises based on the most important SCT constructs. The educational content will be based on the latest national and international guidelines for the prevention of WMSDs (facts). The control group will not receive the studied intervention and will be provided them with information about ways to prevent breast cancer. The HSE expert will communicate with the participants during the intervention to teach them how to maintain a proper body posture at work and remind them to adhere to the intervention, and stretching exercises will be taught to them during breaks. Participants will receive phone calls and messages on social networks from the research team in the 12 weeks after the intervention to improve participation and adherence.

### Recruitment

Before recruiting women, the project researchers will meet with Electronics Industries personnel (e.g., industry managers, and HSE experts) to discuss the project and its requirements. Industries that agree to provide participation opportunities to their workers who are included in the study. We expect to enroll participants consecutively during a 30-day recruitment period. All potentially eligible women will be discussed directly with the aims and potential benefits of the study.

In this study, women are not included if stretching exercises are prohibited by the occupational medicine doctor, so no harm or adverse effects are expected from participating in the trial. However, a general physician (4th authors) was invited to participate in the study. He has responsibility to consider all health aspects of the project and assess all probable medical complications during the study. Moreover, all participants are asked to report any possible side effects to be assessed by physician. The control group will receive the delayed intervention after completing the 6-month follow-up questionnaire.

### Outcomes

The main outcomes in the present study include the final score of body posture at work obtained from the RULA method. The score of adherence to stretching exercises at work.

The secondary outcome includes the following:

- The scores of SCT constructs are measured using a researcher-made questionnaire.
- There is no reliable tool to measure the preventive behaviors of WMSDs among assembly-line female workers based on SCT in Iran; therefore, evaluating the validity of the researcher-made questionnaire is a secondary outcome.
- A researcher-made questionnaire is used to measure percentage of people who regularly perform physical activity, 3, 12, and 24 weeks after the intervention.
- A researcher-made questionnaire is used to measure the percentage of people who consume low-fat dairy products, 3, 12, and 24 weeks after the intervention.

### Sample size

Considering that the average total number of assembly-line female workers is about 500 people, sample size was estimated 217 people using Cochran’s formula and considering the type I error (0.05). However, taking into account the 15% possible dropout, it was estimated 250 people in phase 2.

In addition, according to the study by Amit [43], who similarly used an ergonomic intervention to investigate the body posture and WMSDs in workers, to ensure sufficient power and taking into account 10% dropout, the required sample size of 40 people was calculated for each group in phase 3. This sample size was considered sufficient to test the difference between groups at power (80%) and alpha (0.05).

### Sampling

The industries will be randomly allocated to the control and intervention groups by throwing dice. Then, among the assembly-line female workers in each industrial unit, the eligible ones are determined and 40 participants randomly selected (a list of workers is prepared and based on table of random numbers) from each industry. The randomization unit is the industrial unit. Opaque sealed envelopes will be used to ensure the allocation concealment. The researcher will generate the allocation sequence, enroll participants, and assign them to interventions.

### Blinding

The control group will also be provided with an educational intervention that is not related to the main objective of the research, and since the control and intervention groups are assigned into two separate industries, the workers will be unaware of the group assignment. Since the participants will be assigned a code, data and outcome analysis will be unaware of the names of the participants and the control and intervention groups. The main researcher of the study will not be blinded.

There are no known circumstances in which emergency unblinding would be required; however, if there are compelling reasons, such as if the information is needed for recruitment purposes, the principal investigator will provide permission for unblinding. The protocol deviation form should be registered in the trial database.

Participants’ personal information is assigned a unique identification code, stored in a password-protected format, and accessible only to the principal investigator. Participants cannot be identified in manuscripts, reports, or research-related presentations. Data that will be entered into SPSS will be double checked to promote data quality.

### Instruments

Data collection instruments will include the researcher-made questionnaire based on the SCT constructs, the Nordic questionnaire [44], and the RULA method to assess the risk of upper limb MSDs [45]. The questionnaires will be completed by the two groups before the intervention, 3, 12, and 24 weeks after the educational intervention. Table 3 shows the general characteristics of the aforementioned instruments.

**Table 3:** Summary of the instruments used for data collection

Scale	Content	Scoring
Researcher-designed questionnaire	Structures of social cognitive theory	Totally disagree (1) to totally agree (5)
RULA	Rapid upper limb assessment	1 (no need for assessment) to 4 (immediate assessment is required) level
Nordic	Identifying musculoskeletal disorders	1 yes to no 2

### Data analysis

Data analysis will be performed using intention-to-treat analysis in the SPSS ver. 19 software and Bayesian network model. First, exploratory factor analysis will be conducted to assess construct validity after confirming sampling adequacy based on the KMO and Bartlett’s test statistic. Second, the Bayesian network model will be used to determine the variables affecting the use of preventive behaviors for MSDs.

Third, data distribution will be investigated using the Shapiro–Wilk test. Fisher’s exact test will be used for qualitative data; independent *t*-test or Mann–Whitney test will be used to determine the mean score of the studied constructs in two control and intervention groups. Pearson and Spearman correlation tests will be used for parametric data and Kendall rank correlation for non-parametric data. Moreover, to compare the difference between the values obtained before the intervention and 3, 12, and 24 weeks after the intervention in each group, generalized mixed models of repeated measure analysis of variance will be used. Bonferroni’s correction will be used for multiple primary outcomes. The difference between the means of the independent groups will also be calculated at 95% confidence interval. *P*-value < 0.05 will be considered as the statistically significant level. No replacement of study subjects or imputation of missing data will be carried out.

### Dissemination policy

Trial results will be submitted to peer-reviewed journals for publication. Part of the data, such as information regarding the main outcome, can be shared.

## Discussion

The present study focuses on workplace health promotion by investigating the effect of theory-based ergonomic intervention on the preventive behaviors of WMSDs among assembly-line female workers. The main framework of the present study is a theory-based intervention with the aim of maintaining correct body posture at work and performing stretching exercises using the LBD approach in the workplace. Assembly-line female workers undertake a large amount of family and social duties and responsibilities at the same time [46], and it seems that designing appropriate educational interventions can be helpful in promoting correct ergonomic behaviors in these women.

Studies have suggested a revision in the design of educational interventions in workplaces [47, 48]. An educational model directs the program in the right path and reduces the ambiguities in the main content of the intervention and provides a framework for guidance and evaluation in interventions [49]. In the present study, a theory-based educational intervention will be designed in a structured manner, and the LBD approach will be used for workers in their workplace.

Therefore, the scoring system is based on Bayesian network and fuzzy sets. In other studies, these analyzes have yielded a more accurate evaluation on the effectiveness of ergonomic interventions [20]. To the best knowledge of the author, this is the first study that will evaluate the effects of theory-based educational intervention based on Bayesian model and fuzzy sets. On the other hand, other previous ergonomic interventions on the assembly-line workers have not used health education theories [41, 50].

Due to economic problems in the industrial environment, it will not be possible to carry out physical ergonomic interventions in the workplace, such as changing tables and chairs in the assembly halls or using ergonomic tools. In addition, the intervention will only be carried out on assembly-line female workers. Other limitations of the present study include the use of a self-reporting tool that results will be inevitably affected by the memory error, lack of clarity, and individual biases.

This study will evaluate the effect of theory-based ergonomic intervention on the preventive behaviors of MSDs in Northeast Iran. This theory-based ergonomic intervention strategy with a LBD approach, if reported to be effective, may become an important tool to reduce the burden of MSDs, and subsequent disabilities and absenteeism, and increase productivity and job satisfaction of women working in the industrial environments.

### Trial status

This is the first version of the protocol on 24 January 2023. Recruitment is expected to be completed by August 2023.

## Data Availability

Non-applicable participant-level data will not be shared.

## Abbreviations

MSDS: Musculoskeletal disorders
RULA: Rapid upper limb assessment
LBD: Learning by doing
WMSDs: Work-related musculoskeletal disorders
SCT: Social cognitive theory
HSE: Health, safety, and environment
